# Simple Iron Halides Enable Electrochemically Mediated ATRP in Nonpolar Media

**DOI:** 10.1021/acsmacrolett.3c00570

**Published:** 2023-11-13

**Authors:** Gianluca Gazzola, Andrea Antonello, Abdirisak A. Isse, Marco Fantin

**Affiliations:** Department of Chemical Sciences, University of Padova, Via Marzolo 1, 35131 Padova, Italy

## Abstract

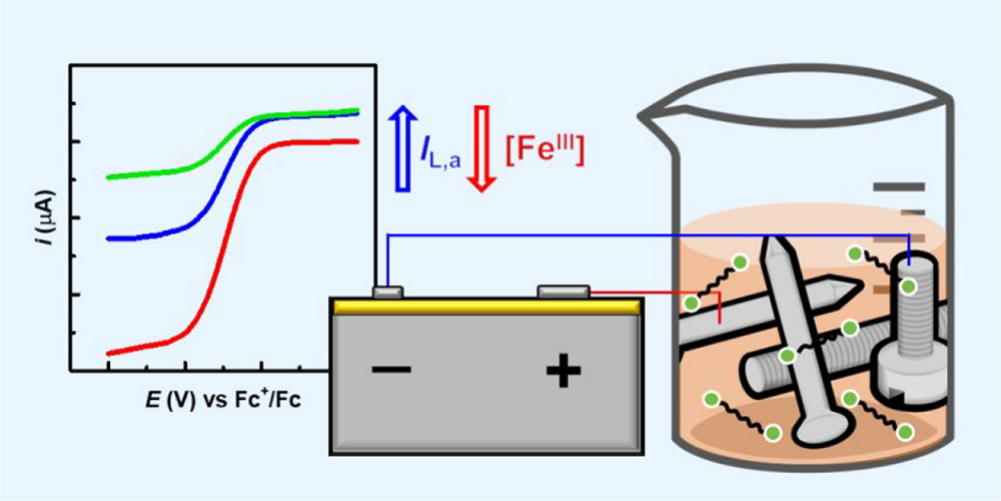

An electrochemically controlled atom transfer radical polymerization (*e*ATRP) was successfully carried out with a minimal amount (ppm-level) of FeBr_3_ catalyst in a nonpolar solvent, specifically anisole. Traditionally, nonpolar media have been advantageous for Fe-based ATRP, but their low conductivity has hindered any electrochemical application. This study introduces the application of electrocatalytic methods in a highly nonpolar polymerization medium. Precise control over the polymerization was obtained by employing anhydrous anisole with only 400 ppm of FeBr_3_ and applying a negative overpotential of 0.3 V. Additionally, employing an undivided cell setup with two simple iron wire electrodes resulted in a significant 15-fold reduction in electrical resistance compared to traditional divided cell setups. This enabled the production of polymers with a dispersity of ≤1.2. Lastly, an examination of kinetic and thermodynamic aspects indicated that the ppm-level catalysis was facilitated by the high ATRP equilibrium constant of Fe catalysts in nonpolar environments.

Reversible deactivation radical polymerization (RDRP) methods are powerful techniques for producing polymeric materials with precisely tailored architectures, low dispersity, and high chain-end functionality.^1^ Atom transfer radical polymerization (ATRP) stands out as one of the most extensively researched and utilized RDRP techniques, owing to its versatility and reliability.^2^

ATRP is based on a reversible halogen atom exchange between a transition metal complex and a growing polymer chain. This dynamic equilibrium between the propagating radical (P_n_^•^) and a dormant species (P-X) guarantees polymerization control and reduces the rate of termination reactions. While Cu remains the most widely employed metal in ATRP, Fe presents several advantages. Its high abundance, environmental friendliness, and lower toxicity^3^ render Fe suitable for potential industrial advancements of ATRP. Furthermore, most Fe catalysts used in ATRP possess very simple structures based on iron halide salts without any additional ligand (L).

The mechanism of iron-catalyzed ATRP is reported in Scheme 1. The Fe complex in its low oxidation state (Fe^II^/L), called activator, reacts with an alkyl halide initiator (RX) or a halogen-capped dormant polymer chain (P_n_-X) to produce a propagating radical (P_n_^•^) and the metal complex in a higher oxidation state with the halogen atom as an additional ligand (X-Fe^III^/L). P_n_^•^ propagates for a short period of time, then it is capped via atom transfer from the deactivator complex X-Fe^III^/L to reform the dormant species P_n_-X. The equilibrium constant, *K*_ATRP_ = *k*_act_/*k*_deact_, defines the equilibrium concentration of propagating radicals and plays a primary role in polymerization kinetics and control over molecular weight distribution (dispersity, *Đ*).^4^

**Scheme 1:**
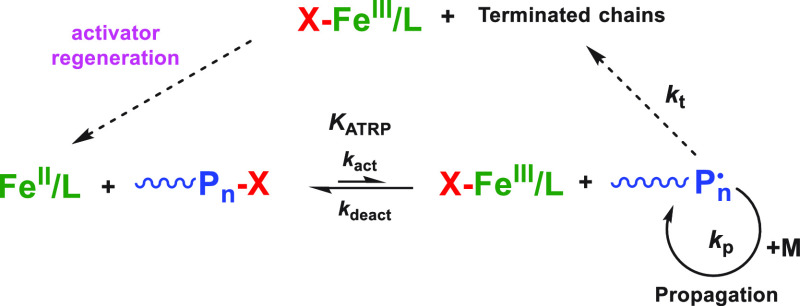
Mechanism of Iron-Catalyzed ATRP with Activator Regeneration

Termination events result in the irreversible accumulation of the deactivator complex, eventually leading to inhibition of the process. Various adaptations of ATRP have been formulated to regenerate the active form of the Fe complex, thereby reducing the catalyst loading. Furthermore, these methods allow starting the process with the metal catalyst in a higher oxidation state, which is resistant to oxygen in comparison to its reduced state. Activators (re)generated by electron transfer (ARGET) ATRP,^5−7^ supplemental activator and reducing agent (SARA) ATRP,^8−10^ initiators for continuous activator regeneration (ICAR) ATRP,^11−13^ photoinduced ATRP,^14−16^ and electrochemically mediated ATRP (*e*ATRP)^17−20^ are some examples of these methods. Each of these techniques offers a more industrially relevant alternative compared to normal ATRP. Notably, *e*ATRP eliminates the need for an external reducing agent, even in continuous-flow setups.^21^ Furthermore, it facilitates the precise regulation of the polymerization rate and offers accurate temporal control.^22,23^

We conceived an electrochemical process in which ppm amounts of iron salts (e.g., FeBr_3_) undergo reduction at an iron working electrode (WE) to regulate the ATRP process. However, these low-ppm processes involving Fe complexes require nonpolar solvents (e.g., bulk monomer or aromatic solvents^14^), which are considered unsuitable for electrochemical syntheses due to their low conductivity. In fact, *e*ATRP procedures developed thus far have employed high-conductivity polar organic solvents (typically DMF), which however demanded a substantial loading of Fe, equimolar to initiator (∼5000 ppm), in order to attain only moderate control over the process.^24,25^

In this study, our objective was to establish an *e*ATRP process within nonpolar environments employing low ppm of Fe catalysts. Furthermore, we aspired to use the most straightforward electrochemical polymerization system feasible, employing merely two mild steel electrodes.

We targeted the *e*ATRP of methyl methacrylate in 50/50 (v/v) anisole/MMA with FeBr_3_ at 400 ppm loading (as [FeBr_3_]/[monomer] × 10^6^, corresponding to 1.88 mM Fe). First, we studied the voltametric behavior of FeBr_3_ in anisole on a glassy carbon (GC) electrode.

Figure 1 shows the cyclic voltammetry (CV) of FeBr_3_ in anisole + 0.2 M *n*-Bu_4_NBF_4_, both in the absence and in the presence of added bromide anions. In the absence of added Br^–^, a broad cathodic peak with a coupled broad anodic peak was observed, indicating the presence of multiple redox species. Indeed, the solvation of FeBr_3_ produces dibromo-, tribromo-, and tetrabromo-iron(III) species, with additional solvent molecules in the coordination sphere.^24^ When a single equivalent of Br^–^ was added, the CV turned into a reversible and well-defined peak couple that was assigned to the Fe^III^Br_4_^–^/Fe^II^Br_4_^2–^ redox couple, with half-wave potential *E*_1/2_ = −0.413 V vs Fc^+^/Fc and peak separation of 92 mV at *v* = 0.1 V s^–1^ (see additional voltammetric data in Figure S1). Further additions of bromide anions did not induce significant modifications in the voltametric response, indicating that they did not further bind to the metal center. This was confirmed by the concomitant increase in the intensity of the anodic peak located at 0.21 V vs Fc^+^/Fc, which is associated with the oxidation of free Br^–^.

**Figure 1:**
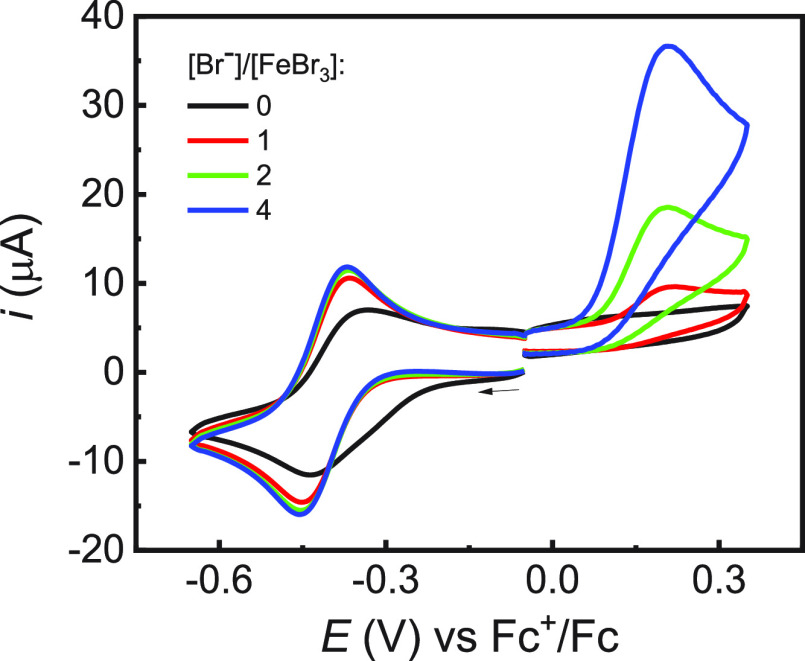
Cyclic voltammetry of 10^–3^ M Fe^III^Br_3_ in anisole + 0.2 M *n*-Bu_4_NBF_4_ recorded on a GC electrode at *v* = 0.2 V/s and *T* = 25 °C, before and after the addition of different amounts of *n*-Bu_4_NBr.

When the reaction medium was changed from pure anisole to anisole/MMA (50/50, v/v), the voltametric pattern of FeBr_3_ did not change, except for a slight negative shift of *E*_1/2_. A well-defined reversible peak coupled with *E*_1/2_ = −0.517 V vs Fc^+^/Fc was observed for the Fe^III^Br_4_^–^/Fe^II^Br_4_^2–^ redox couple (Figure S2).

In summary, these observed redox and speciation characteristics indicate the presence of a well-behaved electrocatalyst for ATRP in anisole. This stands in contrast to the behavior of the same FeBr_3_ catalyst in DMF, where multiple reduction and oxidation peaks were observed even after the addition of several equivalents of *n*-Bu_4_NBr.^14^ Therefore, we next tackled *e*ATRP in nonpolar anisole.

The initial reaction development was carried out in a typical divided-cell setup (Table 1, entries 1–7). *e*ATRPs were driven with a platinum mesh WE, an Ag/AgI reference electrode, and a graphite counter electrode (CE) separated via a glass frit and a methylcellulose gel (this setup is indicated as Pt ⫶ C in Table 1, where the vertical dots denote the separator). The polymerization was started upon application of an overpotential η, defined as *E*_app_ – *E*_1/2_, with reference to the value of *E*_1/2_ of the Fe^III^Br_4_^–^/Fe^II^Br_4_^2–^ couple (Figure S2). At η = −60 mV, a typical value for *e*ATRP, no polymerization was observed (Table 1, entry 1). However, simply the application of a more negative overpotential (η = −340 mV) triggered a controlled radical polymerization, with *Đ* ∼ 1.5 and an experimental molecular weight that matched the theoretical value (Table 1, entry 2). The additional overpotential was required to overcome the electrical resistance of this system.

**Table 1:** *e*ATRP of Methyl Methacrylate (MMA) with FeBr_3_ as Catalyst and EBPA as Initiator in Anisole/MMA (50/50, v/v) + *n*-Bu_4_NBF_4_ Electrolyte^a^

entry	cell setup	η (mV)	electrolyte (M)	[FeBr_3_] (ppm)	solvent	conv (%)^b^	10^4^*k*_p,app_ (min^–1^)^c^	10^–3^*M*_n,GPC_^d^	10^–3^*M*_n,th_^e^	*Đ*^d^
1	Pt ⫶ C	–60	0.2	400	anisole	<5				
2	Pt ⫶ C	–340	0.1	400	anisole	36.4	14.8	7.8	7.5	1.54
3	Pt ⫶ C	–340	0.2	400	anisole	59.7	30.6	11.2	12.1	1.41
4^f^	Pt ⫶ C	–340	0.3	400	anisole	46.4	36.7	9.0	9.4	1.45
5	Pt ⫶ C	–340	0.2	400	dry anisole	69.1	34.1	13.5	13.9	1.38
6	Pt ⫶ C	–340	0.2	200	dry anisole	23.4	7.9	7.8	4.9	1.80
7	Pt ⫶ C	–340	0.2	800	dry anisole	67.7	34.4	15.0	13.7	1.35
8^g^	Pt–Al	–340	0.2	400	dry anisole	41.2	22.6	9.0	8.4	1.19
9	Pt–Fe	–340	0.2	400	dry anisole	60.6	26.7	11.3	12.3	1.24
10	Fe–Fe	–340	0.2	400	dry anisole	75.7	36.0	16.8	15.2	1.19
11	Fe–Fe	–340	0.2	400	anisole + 48 mM H_2_O	20.2	10.8	5.8	4.2	1.21
12	Fe–Fe	–340	0.2	400	anisole + 96 mM H_2_O^h^	<5				

a Other conditions: *V* = 15 mL; [MMA]:[EBPA]:[FeBr_3_] = 200:1:0.08; EBPA = ethyl α-bromophenylacetate; [FeBr_3_]:[*n*-Bu_4_NBr] = 0.08:0.32 when the CE was Fe, [FeBr_3_]:[*n*-Bu_4_NBr] = 0.08:0.08 when CE was Pt; polymerization time = 6 h, *T* = 65 °C.

b Monomer conversion measured by NMR.

c Apparent polymerization rate constant determined as the slope of ln([M]_0_/[M]) vs *t*.

d Determined by GPC.

e Theoretical molecular weight.

f Time of polymerization 3 h.

g No increase in conversion after 4 h.

h Corresponds to a water-saturated solution of anisole.

Next, we optimized the concentration of the supporting electrolyte, which could improve the electrical conductivity of the solution (Table 1, entries 2–4, and Figure S3). Increasing the concentration of *n*-Bu_4_NBF_4_ from 0.1 to 0.2 M led to a doubled polymerization rate and increased control over the process (*Đ* ∼ 1.4). Further increasing the amount of *n*-Bu_4_NBF_4_ did not appreciably benefit the polymerization.

The role of catalyst concentration was also studied (Table 1, entries 5–7). The best results were obtained with 400 ppm of FeBr_3_ (1.88 mM). Lower concentrations resulted in slow and uncontrolled polymerization, likely due to too slow activation/deactivation reactions. Conversely, higher Fe concentrations did not produce any improvement, but introduced a technical problem due to too high electrical current with related high ohmic drop (*iR*), which caused instrumental limitation (i.e., the potentiostat could not provide sufficient voltage between WE and CE to sustain the electrochemical process at the imposed conditions).

Overall, the primary challenge encountered during the *e*ATRP process conducted in a nonpolar medium was the high electrical resistance of the system. This challenge was particularly pronounced when performing the polymerization within a divided cell. In our experimental setup, the cathodic and anodic compartments were separated by a porous glass septum and a methyl-cellulose gel (as shown schematically in Figure 2a). The electrical resistance issue could be significantly alleviated by employing an undivided cell with a sacrificial anode.

**Figure 2:**
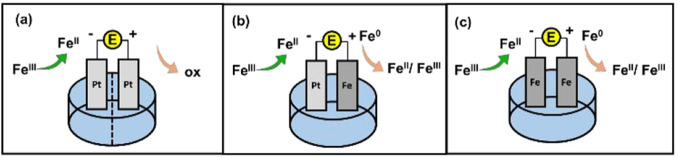
Cell configurations of iron-catalyzed *e*ATRPs performed in this study.

While sacrificial Al anodes are typically used in electrochemical Cu-based ATRP, we discovered that they were incompatible with Fe-based ATRP. This incompatibility likely arose from the interaction between the Fe catalysts and the Al^3+^ ions released from the anode. After a 4 h period, the polymerization using an Al anode ceased, and the solution became cloudy due to the formation of insoluble Al compounds (Table 1, entry 8).

To avoid this contamination issue, we conducted the polymerization with an undivided cell setup comprising a mild steel (iron) wire CE, in combination with a platinum WE and an Ag/AgI RE (Figure 2b). The polymerization exhibited excellent control, following a first-order kinetic rate law, and producing a polymer with narrow dispersity (Figure 3a, red). The oxidation of the iron CE caused the release of Fe^*n*+^ ions in solution (mostly Fe^3+^, Figure S4), which participated in the ATRP equilibrium leading to a better-controlled radical polymerization than those performed in a divided cell setup (Table 1, entry 11 vs entry 7). The amount of released Fe ions from the counter electrode was determined to be only 250 ppm using standard addition methods (Figure S5). The faradic yield of the process was estimated at 41% (see Supporting Information). Most notably, in this undivided cell configuration, the voltage difference (Δ*V*) between the platinum WE and iron CE was only 5 V. This value was 15 times lower than the Δ*V* measured in the divided cell setup, indicating a massive decrease in resistance due to the elimination of the separator.

**Figure 3:**
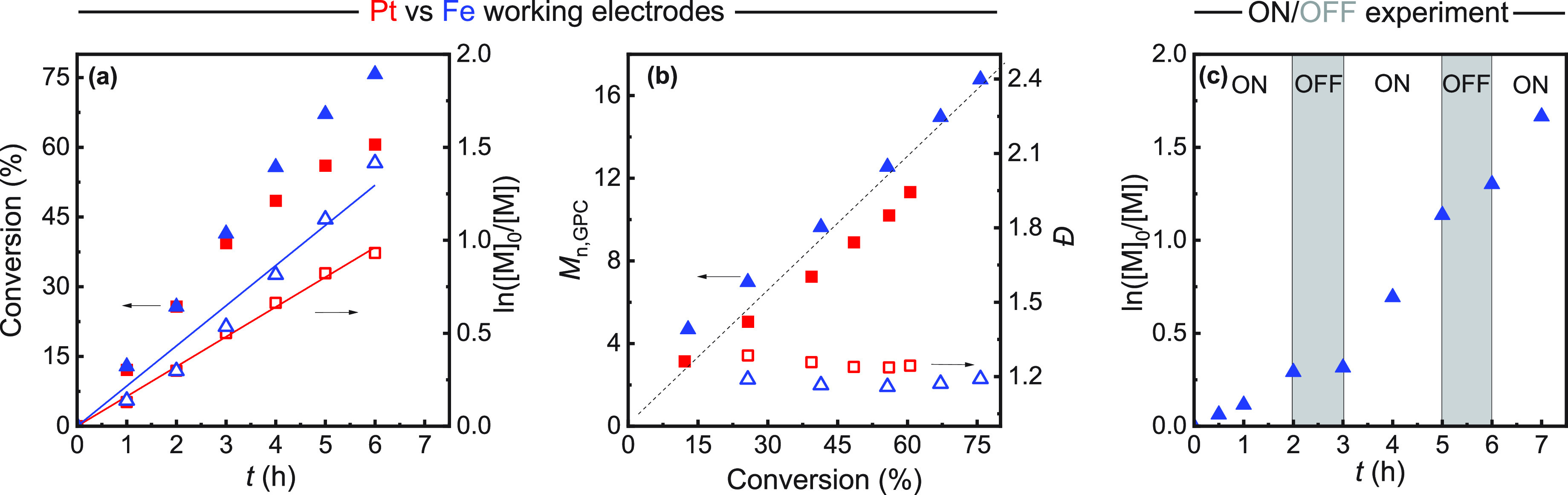
*e*ATRP of MMA in anisole/MMA (50/50, v/v) with Pt mesh (red squares) or iron wire WE (blue triangles). Conditions: [MMA]:[EBPA]:[FeBr_3_]:[*n*-Bu_4_NBr] = 200:1:0.08:0.32; at *T* = 65 °C, performed at *E*_app_ – *E*_1/2_ = −340 mV; 0.2 M *n*-Bu_4_NBF_4_. Undivided cells with a sacrificial Fe anode, Ag/AgI reference electrode. (a) Kinetic plot; (b) Molecular weights and dispersity. The dashed line represents the theoretical *M*_n_. (c) Temporal polymerization control. On iron wire WE and separated CE, the potential was toggled between “ON” (*E*_app_ – *E*_1/2_ = −340 mV) and “OFF” (*E*_app_ – *E*_1/2_ = +500 mV) conditions.

Pt is a very expensive material, which could limit the industrial applications of *e*ATRP. To remove any platinum-group-metal from the system, we explored the use of iron wire as both WE and CE (Figure 2c). The polymerization with this Fe–Fe configuration was faster and presented lower dispersity than the one performed with the Pt–Fe configuration (Table 1, entry 10). Polymerization kinetics exhibited an accelerating trend (Figure 3a, blue), likely due to the release of additional Fe ions into the solution. A control experiment with Fe electrodes but without applied potential showed a slower polymerization that stopped at 27% conversion (Figure S6), likely due to the consumption of the very active EBPA initiator and inefficient comproportionation between Fe^III^ and Fe^0^.

Under electrochemical control, the polymerization could be stopped and restarted by toggling the potential between “ON” (*E*_app_ = *E*_1/2_ – 340 mV) and “OFF” conditions (*E*_app_ = *E*_1/2_ + 500 mV). Since this ATRP system is characterized by a rather high Fe^II^/Fe^III^ ratio, during the “OFF” period a large amount of Fe^II^ was oxidized to stop the process (see below). Figure 3c shows how polymerization quickly stopped after 2 h, upon application of the “OFF” potential on a Fe cathode. The polymerization then promptly restarted after a 1 h “OFF” period. Polymerization was then only partially slowed down after application of “OFF” potential at the 5 h mark, likely due to the impeded mass transport in the viscous solution at high monomer conversions.

Other methacrylates, including benzyl methacrylate and butyl methacrylate (Figures S7 and S8), were polymerized under the conditions in Table 1, entry 10, with great control (*Đ* ∼ 1.2), demonstrating the versatility of this method.

Fe-based ATRP can be influenced by undesired termination reactions, such as reductive radical termination (RRT), which is expedited by incidental water content in the organic solvent (see Scheme S1).^26^ Notably, an augmentation in the water content led to a deceleration in polymerization kinetics, as highlighted in Table 1, entries 5, 11, and 12. In the case of water-saturated anisole (with approximately 96 mM water^27^), no polymerization occurred. Indeed, when anhydrous anisole was used (Table 1, entry 5), the most rapid polymerization, characterized by a linear kinetic plot (Figure S9, blue line), high conversion and low *Đ* was recorded. This indicates a constant radical concentration and the absence of side reactions.

Overall, FeBr_3_ exhibited promising qualities as an electrocatalyst for *e*ATRP in anisole. To understand why the Fe/Br catalysts perform much better in anisole than in DMF, we set out to determine the ATRP equilibrium constant (*K*_ATRP_), along with the rate constants for the forward (*k*_act_) and reverse (*k*_deact_) reactions, for the polymerization of MMA in anisole catalyzed by 400 ppm FeBr_3_. However, this undertaking is challenging for two reasons: (i) the reactivity of Fe catalysts is relatively low (for instance, no catalytic current was detected in cyclic voltammetry in the presence of initiators, as depicted in Figure S2); and (ii) conventional radical traps like TEMPO are not compatible with iron-catalysts for ATRP.^28,29^ Hence, we turned to a novel electrochemical method for determining *K*_ATRP_ under polymerization conditions, employing linear sweep voltammetry (LSV) to monitor the [Fe^III^]/[Fe^II^] ratio throughout an actual polymerization process.

The experiment was performed in the optimal conditions in a divided cell (as in Table 1, entry 5, and Figure S10). Two WEs were present in the cell: a large Pt mesh to reduce the bulk catalyst and trigger polymerization (area ∼10 cm^2^) and a small glassy carbon (GC) electrode to record LSVs (area = 0.07 cm^2^). LSVs of the Fe-based polymerization solution recorded under hydrodynamic conditions, achieved by either magnetic stirring of the solution (Figure 4a) or use of a rotating disk electrode (Figure S11), showed a symmetrical wave with two plateaus, representing anodic (*I*_La_) and cathodic (*I*_Lc_) limiting currents for Fe^II^ oxidation and Fe^III^ reduction, respectively. At *t* = 0, *I*_La_ was zero, confirming that only Fe^III^ was initially present in solution. Ten minutes after the application of the polymerization potential on the Pt mesh WE, a positive *I*_La_ of 1.9 μA was recorded on the GC disk, indicating that Fe^II^ was being generated in solution. As shown in Figure 4a, *I*_La_ increased with time until 30 min, then it tended to decrease. This trend was probably caused by the interplay between Fe^II^ generation (which tends to raise *I*_La_) and the increase in viscosity of the polymerization medium (which tends to lower both *I*_La_ and |*I*_Lc_|). The limiting currents were used to track the catalyst concentration according to the Levich equation (see the Supporting Information). *I*_Lc_ and *I*_La_ are proportional to the instantaneous concentrations of Fe^III^ and Fe^II^, respectively, which were calculated using eqs 2 and 3:

**Figure 4:**
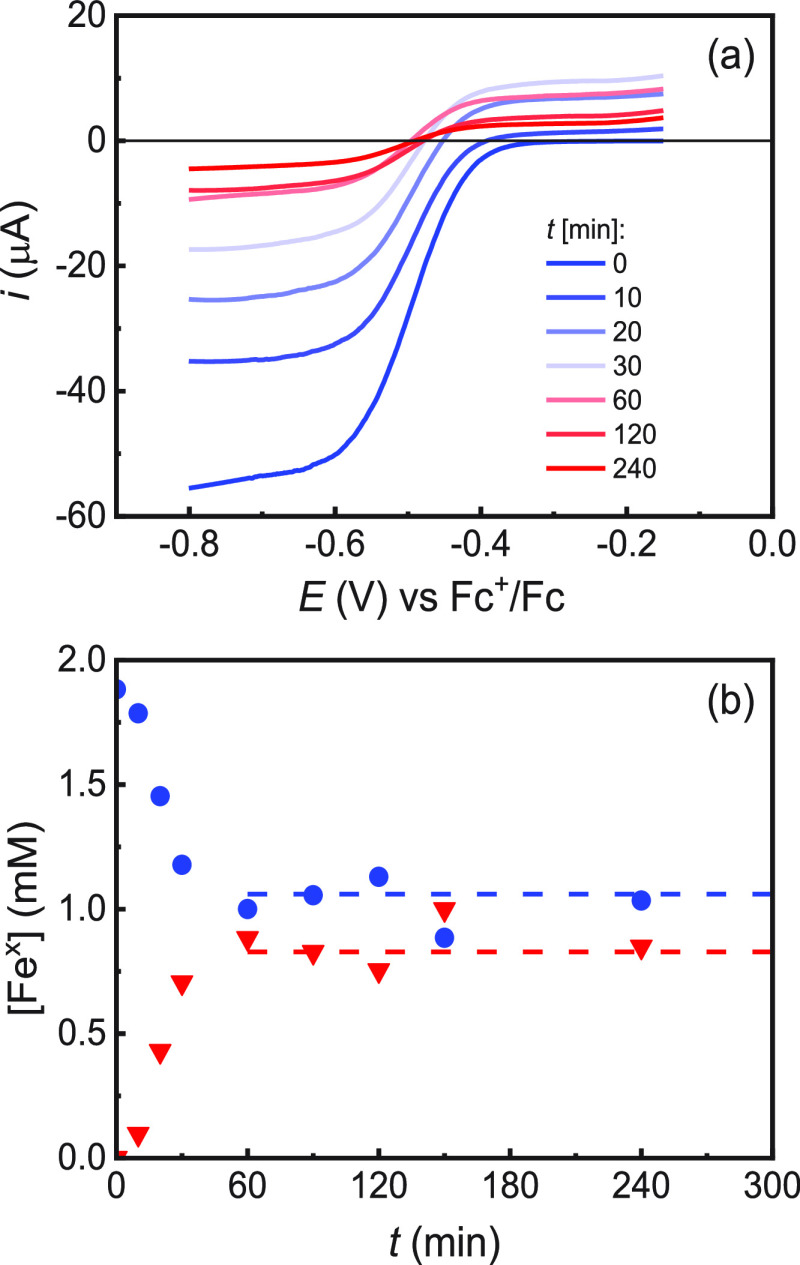
*e*ATRP of MMA in anisole/MMA (50/50, v/v) + 0.2 M *n*-Bu_4_NBF_4_ catalyzed by 400 ppm FeBr_3_ in a divided cell with a Pt mesh WE and graphite CE at *T* = 65 °C. Other conditions: [MMA]:[EBPA]:[FeBr_3_]:[*n*-Bu_4_NBr] = 200:1:0.08:0.08; *E*_app_ = *E*_1/2_ – 0.340 V. (a) LSVs recorded during polymerization; (b) Plots of [Fe^III^] (blue circles) and [Fe^II^] (red triangle) as a function of time. The dashed lines indicate the steady state concentrations after 60 min.


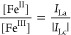
1
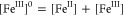
2where  is the initial catalyst loading, which was constant during this *e*ATRP driven on an inert Pt cathode.

Figure 4b shows the plot of the concentrations of Fe^III^ and Fe^II^ vs time. [Fe^III^] and [Fe^II^] are roughly constant after 1 h from the beginning of the polymerization. At the operating potential (*E*_app_ = *E*_1/2_ – 340 mV), the average steady-state value of [Fe^III^]/[Fe^II^] was 1.3. Once the [Fe^III^]/[Fe^II^] ratio during polymerization was known, *K*_ATRP_ was calculated from the ATRP rate law:^4^
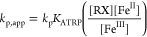
3where *k*_p,app_ is the apparent polymerization rate constant determined as the slope of ln([M]_0_/[M]) vs *t* (see Figure S10) and *k*_p_ is the propagation rate constant for the bulk polymerization of MMA at 65 °C (940 M^–1^ s^–1^).^30^ Additionally, *k*_deact_ could be calculated during the same polymerization process from the values of Fe^III^ concentration and polymer dispersity:
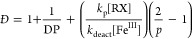
4where DP is the degree of polymerization and *p* is monomer conversion. Finally, *k*_act_ is obtained as *k*_act_ = *K*_ATRP_/*k*_deact_.

For the *e*ATRP of methyl methacrylate in 50/50 (v/v) anisole/MMA mixture at 65 °C, the obtained values are *K*_ATRP_ = 1.9 × 10^–6^, *k*_act_ = 0.44 L mol^–1^ s^–1^, and *k*_deact_ = 2.3 × 10^5^ L mol^–1^ s^–1^ (these values are the averages of two experiments, see Figure S10). These values compare favorably with *K*_ATRP_ = 2.3 × 10^–6^ measured in a similar anisole/MMA system^24^ by spectrophotometric methods. For comparison, the *k*_deact_ in polar *N*-methyl pyrrolidone is similar (8 × 10^5^ L mol^–1^ s^–1^), but *K*_ATRP_ is 2 orders of magnitude smaller (1.4 × 10^–8^).^24^ Due to such low *K*_ATRP_ in dipolar solvents, polymerization requires high Fe loading and is poorly controlled.^19^ The better performance of nonpolar anisole in low-ppm ATRP is due to its higher *K*_ATRP_ that enables a higher Fe^III^/Fe^II^ ratio and therefore higher deactivator concentration and better polymerization control.

In summary, we have reported *e*ATRP catalyzed by low ppm amounts of iron complexes. Electrochemical polymerization was carried out successfully in a nonpolar environment (anisole/monomer solutions). The process required the application of a negative overpotential (η = −340 mV) to compensate for the large Δ*V* drop of the system. The process was expedited by employing anhydrous solvents and increasing the loading of supporting electrolytes. Using an undivided cell setup with iron electrodes resulted in rapid and well-controlled radical polymerizations, reducing the electrical resistance of the system by 15-fold compared to a divided cell setup. This reduction in electrical resistance is a crucial factor for electrochemistry in nonpolar environments. We believe that these findings have significant potential for advancing the utilization of environmentally friendly electrochemical methodologies in nonpolar environments, which encompass numerous green solvents,^31^ as well as the majority of commercially relevant monomers, polymers, and their solutions.

## Supporting Information Available

Materials, methods, additional characterization data (CVs, LSVs), reaction schemes, and polymerization results (PDF)
