# Supplementary material for: Simple Iron Halides Enable Electrochemically Mediated ATRP in Nonpolar Media
Source: ACS Macro Lett. 2023 Nov 13;12(12):1602–7. doi: 10.1021/acsmacrolett.3c00570 (PMC10734308; doi:10.1021/acsmacrolett.3c00570)
Supplement: Supplementary file 1 — mz3c00570_si_001.pdf [file mz3c00570_si_001.pdf]

Supporting information for

Simple Iron Halides Enable Electrochemically-Mediated ATRP in Nonpolar Media

Gianluca Gazzola, Andrea Antonello, Abdirisak A. Isse, and Marco Fantin*

Department of Chemical Sciences, University of Padova, Via Marzolo, 35131 Padova, Italy

S1. Materials and instrumentation

1.1 Material

Anisole used as solvent in electrochemical investigations and polymerizations was purchased from Sigma-Aldrich (99.7%, anhydrous and 99%, not anhydrous) and used without further purification. $FeBr_3$ (Sigma-Aldrich, 98%), $CDCl_3$ (Sigma-Aldrich, 99.8%, anhydrous), LiBr (Alfa Aesar, 99%), basic aluminum oxide (Al_2O_3, Honeywell Riedel-de Haën), DMF (Carlo Erba, 99.9%, HPLC), ethanol (Carlo Erba, 99.8%), ethyl ether (Sigma-Aldrich, 98%), ethyl α-bromophenylacetate (EBPA, Alfa Aesar, 97%), H_2SO_4 (Fluka, 95 %, TraceSELECT), were used as received. Tetraethylammonium tetrafluoroborate (Et_4NBF_4, Alfa Aesar, 99%), used for the preparation of the conductive methyl cellulose gel separating the graphite counter electrode from the polymerization solution in divided cells, was recrystallized twice from ethanol. Tetrabuthylammonium tetrafluoroborate (n-Bu_4NBF_4, Sigma-Aldrich, 99%), used as supporting electrolyte, was recrystallized in double-distilled water. Tetrabuthylammonium bromide (n-Bu_4NBr, Acros Organics, 99%), used a source of Br^- ions in solution, was recrystallized from ethanol/ethyl ether. After recrystallization, all these three salts were dried in a vacuum oven at 80 °C for 48 h. Tetrabuthylammonium iodide (n-Bu_4NI, Sigma-Aldrich, 99%), used for the preparation of the reference electrode, was used without further purification. Methyl methacrylate (Sigma-Aldrich, >99%), benzyl methacrylate (Sigma-Aldrich, 96%) and butyl methacrylate (Sigma-Aldrich, 99%) were percolated through a column of active basic aluminum oxide to remove polymerization inhibitors.

1.2 Instrumentation

Electrochemical measurements were carried out in a 7-neck glass cell under an Ar atmosphere; an Autolab PGSTAT 30 or 30N potentiostat/galvanostat (EcoChemie, The Netherlands) run by a PC with GPES or NOVA software (EcoChemie) was used. The working electrode, counter electrode and reference electrode used during voltammetric investigations (either CV and LSV) were a 3 mm diameter GC disk (Tokai GC-20), a Pt ring and Ag/AgI/0.1 M n-Bu_4NI in DMF, respectively. Before each experiment, the GC disk was cleaned by polishing with a 0.25-µm diamond paste, followed by ultrasonic rinsing in ethanol for 5 min. The reference electrode was always calibrated with ferrocene (Fc), which was added at the end of each experiment as an internal standard, and all potentials are reported versus the ferrocenium/ferrocene (Fc^+/Fc) redox couple. Compensation of resistance was applied during all CV experiments (ca. 80% resistance compensated), unless otherwise noted. No compensation was applied during electrosynthesis (*e*ATRP) experiments.

*e*ATRP experiments were carried out with a Pt mesh (Alfa Aesar, 99.9 % metals basis, area = ca. 10 cm^2, unless otherwise noted) working electrode or an iron wire (Sigma-Aldrich, 99%) and the same reference electrode used in cyclic voltammetry. Before each experiment, the Pt mesh was electrochemically activated in 0.5 M H_2SO_4 by cycling the potential from -0.7 V to 1 V vs Hg/Hg_2SO_4 at a scan rate of 0.2 V s^{-1} (60 cycles). For the polymerizations carried out in a divided cell, a graphite rod was used as counter electrode. The rod was separated from the working solution by a glass frit filled with a solution of Et_4NBF_4 0.1 M in DMF and a methylcellulose gel saturated with Et_4NBF_4. For the polymerizations carried out in an undivided cell, iron wire and aluminum wire were used as electrodes without any activation step. Metal wire electrodes were 12 cm long, for a total area of 6 cm^2.

Gel permeation chromatography (GPC) was used to determine the number average molecular weight (M_n) and dispersity ($Đ$) of polymers prepared by *e*ATRP. The GPC instrument was Agilent 1260 Infinity, equipped with a refractive index (RI) detector and two PLgel Mixed-D columns (300 mm, 5 µm) connected in series. The column compartment and RI detector were thermostated at 70 °C and 50 °C, respectively. The eluent was DMF containing 10 mM LiBr, at a flow rate of 1 mL/min. Before injection, the samples were filtered through alumina over a PTFE membrane of 200 nm pore to remove any particulate material and the iron catalyst. The column system was calibrated with 12 linear poly(methyl methacrylate) standards (M_n = 540-2210000). Monomer conversion was determined by ^{1}H-NMR spectroscopy with a 200 MHz Bruker Avance instrument, using $CDCl_3$ as a solvent.

UV-Vis spectra were recorded with an Agilent Cary 5000 spectrophotometer by using 10 mm optical path length quartz cuvettes.

S2. Methods

S2.1 eATRP

A thermostated 7-neck electrochemical cell, flushed with an inert gas, was loaded with anisole/MMA (50:50, v/v) + 0.2 M *n*-Bu_4NBF_4, the desired amount of iron catalyst and *n*-Bu_4NBr. After recording a CV of the catalyst, the initiator EPBA was injected, and another CV was recorded. Polymerization was then started by applying the selected applied potential (E_{app}), and samples were withdrawn periodically to measure monomer conversion, and M_n and *Đ* of the polymer.

S2.2 Estimation of $[Fe^{III}]/[Fe^{II}]$ during eATRP

To determine K_{ATRP}, k_{act} and k_{deact}, the evolution of Fe^{III} and Fe^{II} concentrations was monitored during a typical *e*ATRP experiment in a divided cell a Pt WE and a graphite CE. This was done by periodically stopping electrolysis and immediately recording a linear sweep voltammogram under stirring to create steady state conditions. As expected, well-defined waves, showing both anodic and cathodic limiting currents, were observed (see Fig. 4, main text). The anodic and cathodic limiting currents, I_{La} and I_{Lc}, respectively, are given by:[1]

$$I_{\mathrm{La}} = nFAm_{\mathrm{Fe^{II}}}[\mathrm{Fe^{II}}] \qquad \text{(S1)}$$

$$I_{\mathrm{Lc}} = -nFAm_{\mathrm{Fe^{III}}}[\mathrm{Fe^{III}}] \qquad \text{(S2)}$$

where *n* is the number of exchanged electrons, *F* is Faraday's constant, *A* is the area of the electrode, and $\mathrm{m_{Fe^{II}}}$ and $\mathrm{m_{Fe^{III}}}$ are mass-transfer coefficients of $\mathrm{Fe^{II}Br_4^{2-}}$ and $\mathrm{Fe^{III}Br_4^{-}}$, respectively. Dividing S1 by S2, with the assumption that the two Fe species have approximately the same mass-transfer coefficient, we obtain:

$$\frac{I_{\mathrm{La}}}{|I_{\mathrm{Lc}}|} = \frac{[\mathrm{Fe^{II}}]}{[\mathrm{Fe^{III}}]} \qquad \text{(S3)}$$

Combining the above equation with the mass balance for iron, $[\mathrm{Fe^{III}}]_0 = [\mathrm{Fe^{III}}] + [\mathrm{Fe^{II}}]$, enables to calculate the concentration of both iron species present in solution.

S3. Characterization data (CVs, LSVs), reaction schemes and polymerization results.

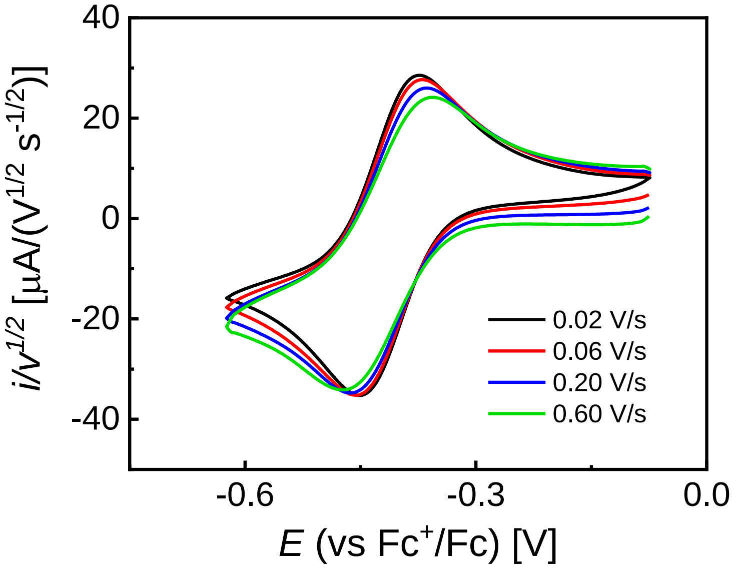

Figure S1. Cyclic voltammetry of 1 mM $Fe^{III}Br_3$ in anisole + 0.2 M n-Bu_4NBF_4 + 4 mM n-Bu_4NBr, recorded on a GC electrode at different scan rates at T = 25 °C.

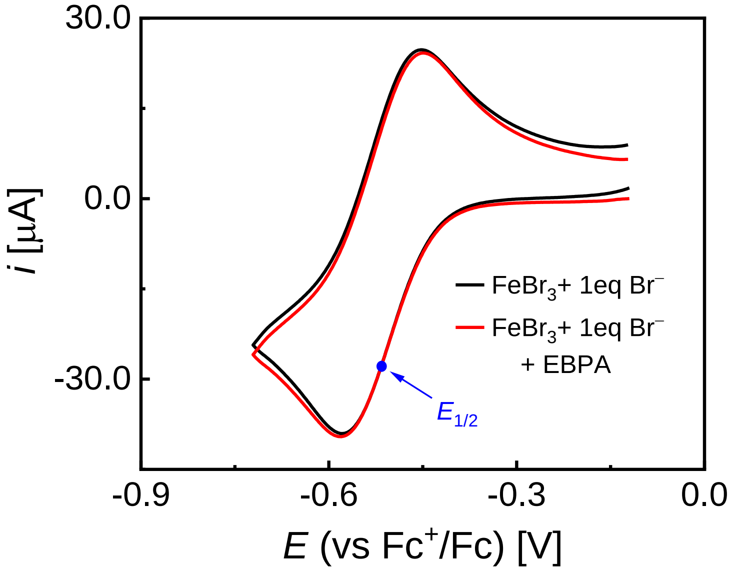

Figure S2. Cyclic voltammetry of 1.88 mM $Fe^{III}Br_3$ in anisole/MMA (50/50, v/v) + 0.2 M n-Bu_4NBF_4 + 1.88 mM n-Bu_4NBr recorded on a GC electrode at v = 0.2 V/s and T = 65 °C, in the absence (black line) and presence of 23.6 mM EBPA (red line).

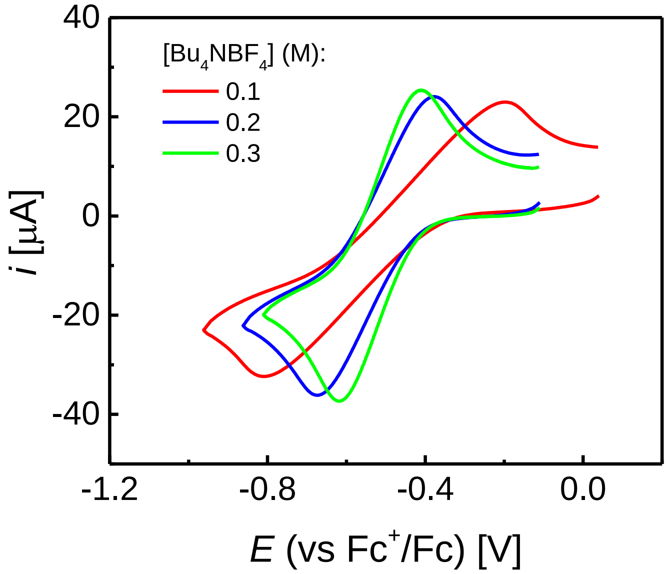

Figure S3. Cyclic voltammetry of 1.88 mM $Fe^{III}Br_3$ in anisole/MMA (50/50, v/v) + n-Bu_4NBF_4 + 1.88 mM n-Bu_4NBr recorded on a GC electrode at v = 0.2 V/s and T = 70 °C, with different supporting electrolyte concentrations and without any compensation of the ohmic drop (iR).

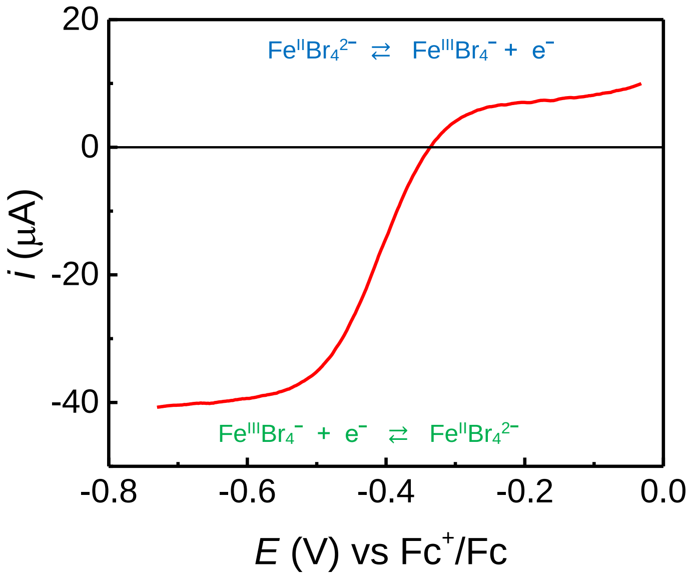

Figure S4. Linear sweep voltammetry (LSV) of Fe^X ions generated after a two-hour galvanostatic oxidation (i = 1.13 mA) of an iron wire in anisole + 0.2 M n-Bu_4NBF_4 + 7.52 mM n-Bu_4NBr, recorded on a GC rotating disk electrode at v = 0.01 V/s, ω = 1000 rpm and T = 65 °C. V_{tot} = 15 mL. The $[Fe^{II}]/[Fe^{III}]$ ratio can be estimated as explained in section S2.2.

S4. Determination of the final concentration of iron and characterization of the process

Final iron concentration ($C_{Fe^{III}}^{6h}$) in polymerization solution (Table 1, entry 9 of the main text) has been determined by spectrophotometric titration of Fe^{III} (50 mM in anisole) with an Agilent Cary 5000 spectrophotometer and 10 mm optical path length quartz cuvette, using the standard addition method. To prepare the sample for analysis, the polymerization media was diluted 50 times (1:49) in anhydrous anisole. An UV-VIS spectrum of this solution was recorded (Figure S5). Then, standard addition of various known aliquots of $FeBr_3$ was performed using a 200 µL micropipette. A UV-Vis spectrum was recorded after every addition, and the final catalyst concentration was calculated from the linear equation obtained by fitting the experimental absorbance at 475 nm (S4). The final Fe concentration in the polymerization solution, after considering the 50x dilution, was determined as 3.25 ± 0.07 mM (corresponding to a release of 1.23 mM additional Fe). This final concentration represents a 65% increase from the initial concentration of 1.88 mM.

$$y = 0.488 + 7.343x \quad \text{(S4)}$$

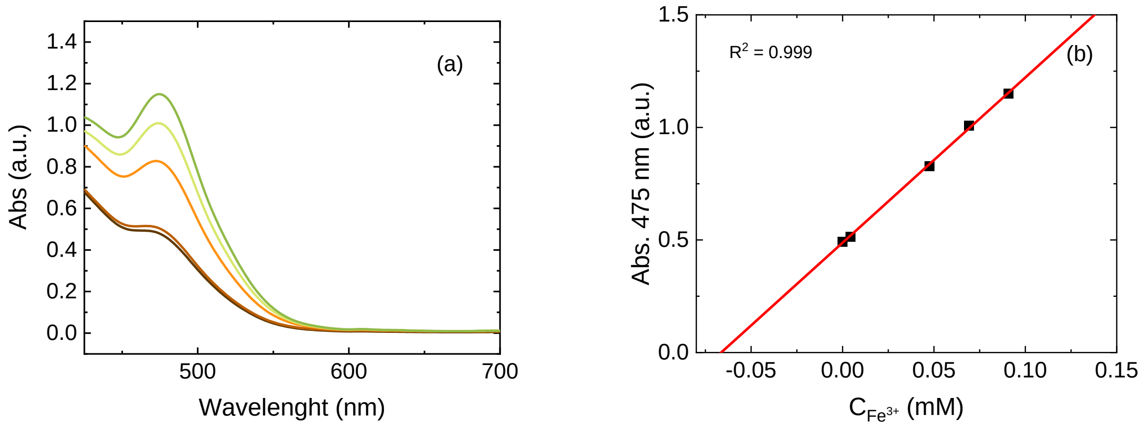

Figure S5. (a) UV-vis spectra of the polymerization solution diluted 50 times (black line) and of subsequent addition of 10, 100, 50, 50 µL stock solution; (b) linear fit of the absorbance registered at 475 nm.

Estimation of Faradaic efficiency for process with sacrificial anode

Using a sacrificial Fe anode setup within a non-separated cell configuration, two reactions could occur at the anode. First, the oxidation of the Fe surface takes place, whereby Fe^{3+} is liberated into the polymerization solution as per half-reaction (S5). Second, $Fe^{II}Br_4^{2-}$ (produced at the cathode can reach the anode and undergo oxidation), as described in equation (S6). This last reaction represents a "parasitic" cycle whereby $Fe^{II}Br_4^{2-}$ is unproductively cycled between the two electrodes (effectively "shorting" the system). These cyclic processes are unproductive but inevitable when using a non-separated cell configuration. Overall, reaction S6 lowers the faradic yield for the generation of new Fe ions from the sacrificial anode.

$$Fe^0 \rightleftarrows Fe^{3+} + 3e^- \quad \text{(S5)}$$

$$Fe^{II}Br_4^{2-} \rightleftarrows Fe^{III}Br_4^- + e^- \quad \text{(S6)}$$

The faradic yield is computed as follows. Given the initial and final concentrations of iron ($C_{Fe}^{t=0}$ and $C_{Fe}^{t=6h}$), the theoretical charge required to produce 1.23 mM of catalyst in solution previously calculated by titration (Q_{th}) can be obtained as follows:

$$Q_{th} = nFV\left(C_{Fe}^{t=6h} - C_{Fe}^{t=0}\right) = 5.3\ C \quad \text{(S7)}$$

The total consumed charge during the 6-hour polymerization process was $Q_{exp} = 12.8\ C$. The Faradic yield was therefore: $\frac{Q_{th}}{Q_{exp}} = 41\%$

S5. Quantification of ohmic resistance with different setups

During an electrochemical process, the potential difference (ΔV) existing between the CE and the WE arises from the combination of two distinct contributions (Equation S9): *i)* a potential drop due to the solution resistance (iR); *ii)* a contribution stemming from the potentials of the semi-reactions at the two electrodes (ΔE), including overpotentials related with the electrode reactions (e.g. activation and mass transfer overpotentials). A high cell resistance, particularly when coupled with the utilization of a low-polarity polymerization medium, proves disadvantageous in the context of bulk electrolysis processes. A substantial ohmic component translates to dissipated energy as heat.

$$\Delta V = iR + \Delta E \qquad \text{(S9)}$$

In the configuration employing a separate-cell CE (as employed in experiments 1 to 7 of the main text), the ΔV recorded at the onset of the reaction was approximately 90 V. Given that ΔE $\ll iR$, we can estimate the cell resistance by approximating $\Delta V = iR$. Accounting for an initial current of 1.2 mA during the initial stages of polymerization the system resistance is 75 KΩ.

A setup employing sacrificial Fe anode was used to obviate the resistance associated with the presence of the porous septum dividing the anodic and cathodic compartments. The ΔV existing between CE and WE in this setup was approximately 5 V. Calculated resistance in this case, ignoring again ΔE in equation S9, the maximum solution resistance is 5 KΩ. The sacrificial anode configuration thus enables overall energy saving, even in the presence of useless cycles (Equation S6), owing to the diminished system resistance. Moreover, in the lab scale this setup remains applicable with inexpensive potentiostats with low potential compliance.

S6. SARA ATRP with Fe wire

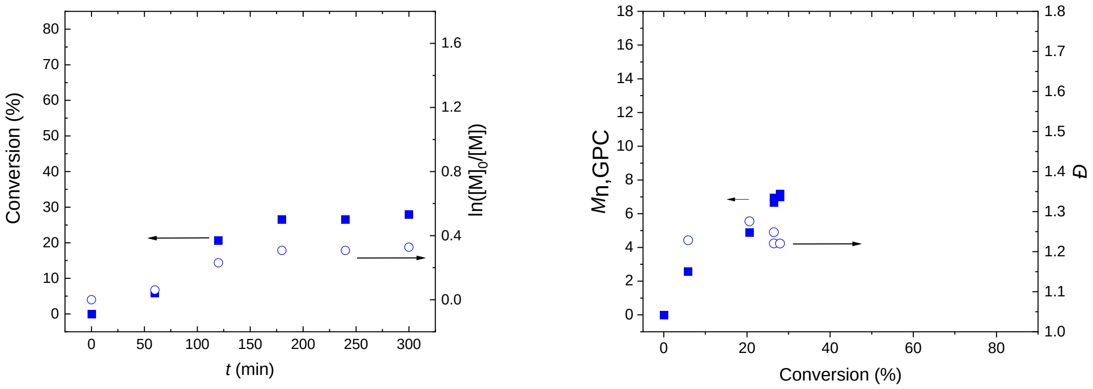

Figure S6. Control experiment for eATRP of MMA mediated by 1.88 mM (400 ppm) $FeBr_3$ in anhydrous anisole/MMA (50/50, v/v) + 0.2 M *n*-Bu_4NBF_4 + 3.60 mM *n*-Bu_4NBr + 14.8 mM EBPA, performed in a cell with an Fe-Fe setup without any applied.

S7. Polymerization of benzyl methacrylate and butyl methacrylate

Table S1. *e*ATRP of benzyl methacrylate (BzMA) and butyl methacrylate (BMA) with $FeBr_3$ as catalyst and EBPA as radical initiator in anisole/monomer (50/50, v/v) + 0.2 M *n*-Bu_4NBF_4.

Entry	Cell Setup	$E_{app} - E_{1/2}$ (mV)	EBPA (mM)	[$FeBr_3$] (ppm)	Monomer	Conv (%)[a]	10^4 $k_{p,app}$ (min^{-1})	10^{-3} $M_{n,GPC}$	10^{-3} $M_{n,th}$	$Đ$
1	Fe - Fe	-340	14.8	400	BzMA	34.2	12.0	10.9	11.9	1.21
2	Fe - Fe	-340	15.7	400	BMA	76.4	41.0	14.4	16.5	1.15

Other conditions: WE = iron wire, CE = iron wire, V = 15 mL; [BzMA] = 2.95 M; [BMA] = 3.14 M; DP = 200; [$FeBr_3$]:[n-Bu_4NBr] = 1:3. [a]Conversion after 6 h of polymerization for BzMA and 5 h for BMA.

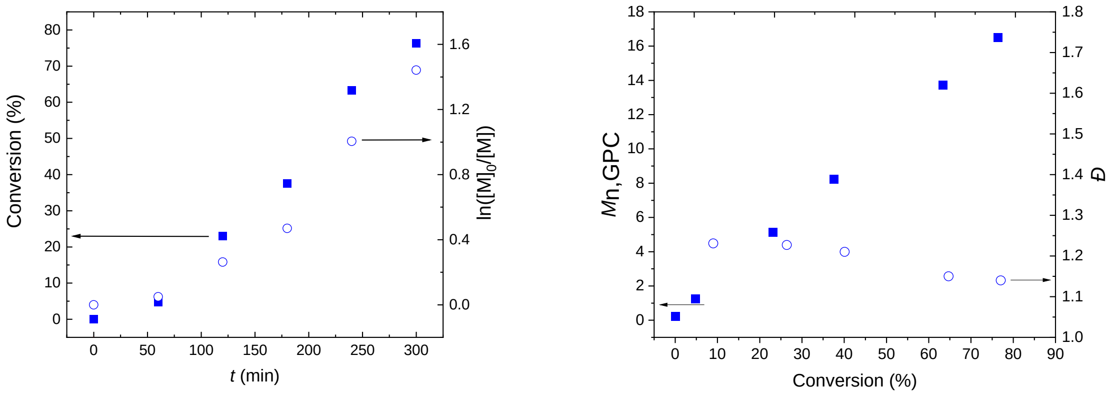

Figure S7. eATRP of butyl methacrylate mediated by 1.26 mM (400 ppm) $FeBr_3$ in anhydrous anisole/BMA (50/50, v/v) + 0.2 M n-Bu_4NBF_4 + 3.78 mM n-Bu_4NBr at T = 70°C, with 15.7 mM EBPA as initiator at $E_{app} - E_{1/2}$ = -340 mV.

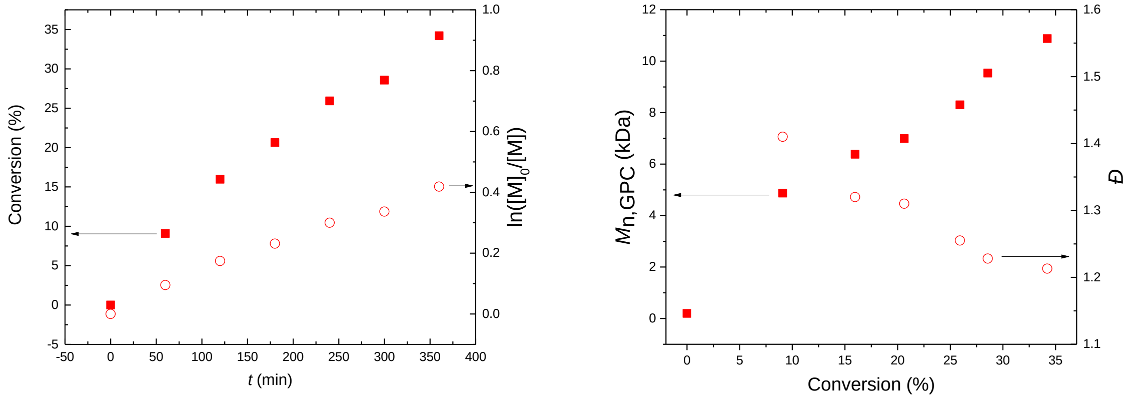

Figure S8. eATRP of BzMA mediated by 1.20 mM (400 ppm) $FeBr_3$ in anhydrous anisole/BzMA (50/50, v/v) + 0.2 M n-Bu_4NBF_4 + 3.60 mM n-Bu_4NBr at T = 70°C, with 14.8 mM EBPA as initiator at $E_{app} - E_{1/2}$ = -340 mV.

Scheme S1. Mechanism of reductive radical termination (RRT).

$$P_n^{\bullet} + Fe^{II}L \longrightarrow P_n - Fe^{III}L$$

$$P_n - Fe^{III}L \xrightarrow{HX} X - Fe^{III}L + P_n - H$$

S8. Polymerization with different grades of anisole

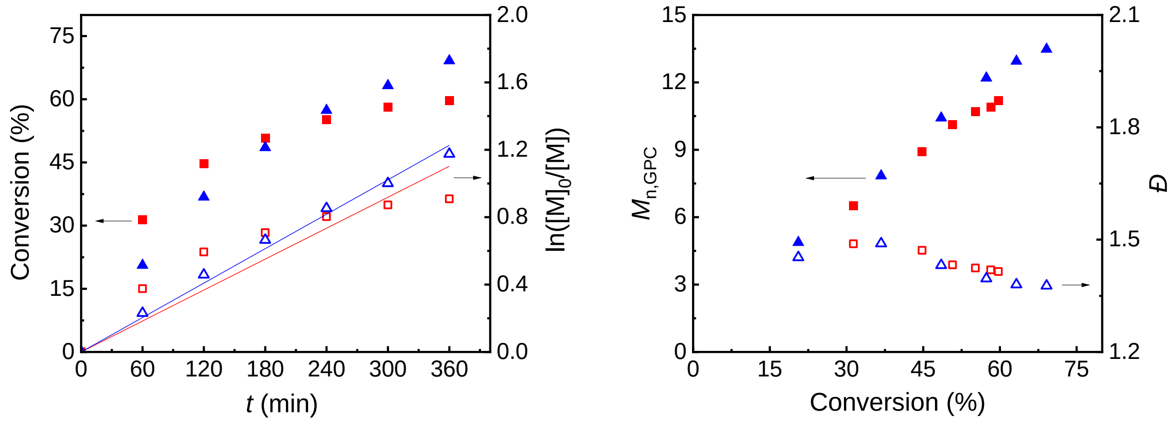

Figure S9. *e*ATRP of MMA mediated by 1.88 mM $FeBr_3$ in anisole/MMA (50/50, v/v) + 0.2 M *n*-Bu_4NBF_4 + 1.88 mM *n*-Bu_4NBr at T = 65 °C, with 23.6 mM EBPA as initiator at $E_{app} - E_{1/2}$ = -340 mV. Anisole (red squares) and anhydrous anisole (blue triangles).

S9. Polymerization for the calculation of kinetic and thermodynamic values

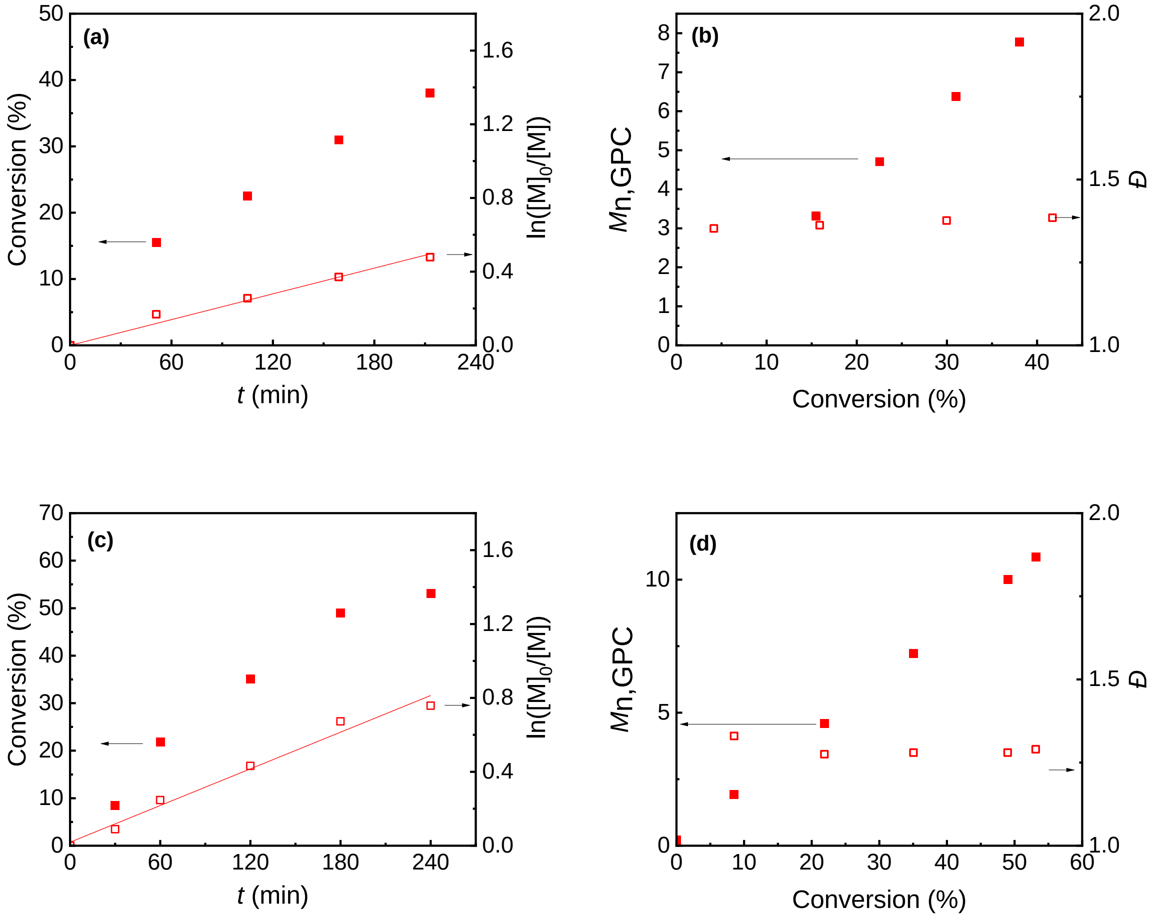

Figure S10. eATRP of MMA mediated by 1.88 mM $FeBr_3$ in anisole/MMA (50/50, v/v) + 0.2 M *n*-Bu_4NBF_4 + 1.88 mM *n*-Bu_4NBr at T = 65 °C, with 23.6 mM EBPA as initiator at $E_{app} - E_{1/2}$ = -340 mV. The reaction was carried out in the presence of two working electrodes, a large Pt mesh to reduce the bulk Fe^{III} to Fe^{II}, and a small GC disk to record linear sweep voltammograms (LSV). The electrolysis on the large mesh was stopped, and immediately a LSV was recorded. Then, the polymerization was restarted. (a, c) Kinetic plots. The slight difference in kinetic rate is due to a different size of Pt mesh. (b, d) M_n and dispersity. Hydrodynamic conditions achieved by either (a, b) fast magnetic stirring or (c, d) rotating disk electrode (2000 rpm).

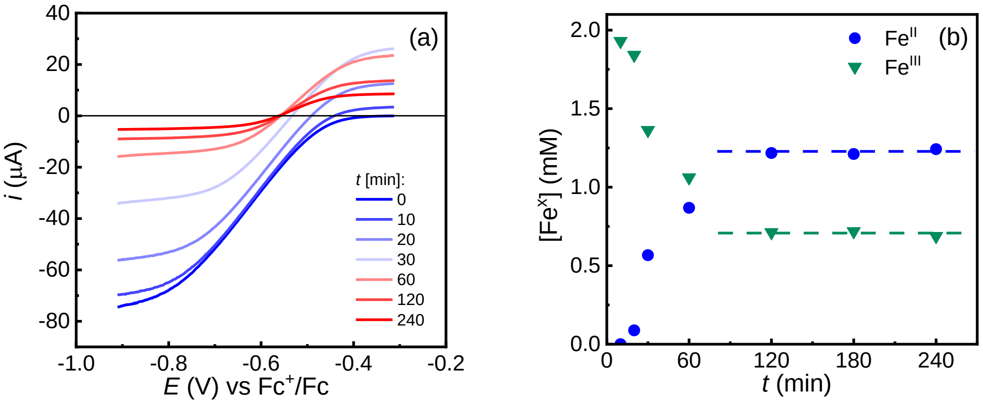

Figure S11. *e*ATRP of MMA in anisole (50/50, v/v) + 0.2 M *n*-Bu_4NBF_4 catalyzed by 400 ppm $FeBr_3$ in a divided cell with a Pt mesh WE and graphite CE at T = 65 °C. Other conditions: [MMA]:[EBPA]:[$FeBr_3$]:[*n*-Bu_4NBr] = 200:1:0.08:0.08; $E_{app} = E_{1/2} - 0.340$ V. (a) LSVs recorded at v = 5 mV/s on Pt RDE ω = 2000 rpm during polymerization; (b) plots of [Fe^{III}] (blue circles) and [Fe^{II}] (green triangle) as a function of time. The dashed lines indicate the steady state concentrations after 60 min. Values for kinetics constants can be determined as described in section S2.2. The obtained values are $K_{ATRP} = 1.44\times10^{-6}$, $k_{act} = 3.31\times10^{-1}$ L mol^{-1} s^{-1}, and $k_{deact} = 2.29\times10^{5}$ L mol^{-1} s^{-1}. The values obtained with RDE in hydrodynamic conditions are comparable to those obtained with the magnetic stirred solution.

References

(1) Bard, A. J.; Faulkner, L. R.; White, H. S. *Electrochemical methods: fundamentals and applications*; John Wiley & Sons, 2022.
